# Rhabdomyolysis due to a hypernatremia hyperosmolar state in trauma brain injured patient: A case report

**DOI:** 10.1016/j.tcr.2025.101283

**Published:** 2025-11-21

**Authors:** L. Bennis, Y. Elouardi, I.E. Raihani, I. Oussayeh, M. Khallouki

**Affiliations:** Anesthesia and Intensive Care Department, Faculty of Medicine and Pharmacy, Cadi Ayyad University, Marrakech, Morocco

**Keywords:** Hypernatremia, Hyperosmolar state, Rhabdomyolysis, Brain injury, Renal failure

## Abstract

Rhabdomyolysis is a serious entity that can progress to acute renal failure and can be life-threatening. Trauma is the most common cause of rhabdomyolysis. However, other etiologies have been reported, including metabolic disorders. We report a case of isolated trauma brain injured patient, who presented a rhabdomyolysis secondary to a hyperosmolar state due hypernatremia, with renal failure and hyperkalemia. To our knowledge, hypernatremia responsible for a hyperosmolar state is a rare cause of rhabdomyolysis.

## Introduction

Rhabdomyolysis is a clinical and biological syndrome secondary to lysis of skeletal muscle cells with release of their contents into the circulation [1]. This lysis may be responsible for renal failure with hyperkalemia, secondary to the direct toxicity of the released myoglobin and its precipitation in the renal tubules, thus threatening the vital prognosis in the short and medium term [[2], [3], [4]]. While trauma remains the etiology most frequently found during rhabdomyolysis, other etiologies have been seldom reported, notably hyperosmolar states [5,6]. We report the case of a traumatic brain injured patient who presented with rhabdomyolysis following a hyperosmolar state due to hypernatremia.

## Clinical presentation

A 47-year-old patient with no past history was admitted to the emergency room for severe trauma following a road traffic accident. Examination revealed an unconscious patient with GCS 7/15, unilateral mydriasis, with heart rate of 122 beat/min, blood pressure of 95/52 mmHg and SaO2 88% on ambient air. No signs of seizures were reported at the scene, and physical examination revealed no signs of crushing or muscle strain.

Initial conditioning was achieved with norepinephrine, orotracheal intubation and deep sedation with midazolam and fentanyl infusion. Then, the patient underwent a body scan, which revealed right temporal and brainstem foci of hemorrhagic contusions with subarachnoid hemorrhage of the brain scythe, parietal and temporal cortical sulci, all associated with significant cerebral oedema. CT-scan did not reveal any other lesions.

As the mydriasis persisted, 0,5g/kg of Mannitol 20% mannitol was given. Osmotherapy was successful, and the patient was transferred to the intensive care unit.

In intensive care, transcranial doppler was normal. Blood tests were as the following: pH at 7,39; PaCO2 at 38mmHg; HCO3 at 24 mmol/L; PaO2 at 165mmHg; Hemoglobin at 11g/dL and Natremia at 146 mmol/L. Thus, neuro-resuscitation measures were continued for 48h.

On day 3 of admission, a fever was presented by the patient related to a pulmonary infection. The biological assessment found white blood cells at 18000/mm^3^, CRP at 130 mg/l, hypernatremia at 165 mmol/L, plasma osmolarity at 343 mOsm/L, urinary osmolarity at 800 mOsm/L, natriuresis at 80 mmol/L. The infection was controlled with antibiotics, and a hydration in plain water by nasogastric tube of dextrose 2.5% was started.

On day 4 of admission, the patient began to develop oliguria and reddish-brown (tea-colored) urine (Picture I), with a biological assessment revealing the worsening of the hypernatremia becoming at 174 mmol/L, a plasma osmolarity at 367 mOsm/L, hypokalemia at 3 mmol/L, urea at 0.8 g/L and creatinine at 13 mg/L. Suspecting rhabdomyolysis as a result of a hyperosmolar state, the correction of hypernatremia was continued and a CPK level was requested, returning to 2500 IU and then 16500 IU (Fig. I). Despite intensive support treatment, the evolution on day 7 was marked by the onset of acute renal failure (urea at 1,18 g/L and creatinine at 33 mg/L) with anuria and hyperkalemia at 6 mmol/L, hence hemodialysis was started. The patient underwent a total of 4 sessions, after which diuresis was resumed and CPK levels and renal function normalized in the following days. Despite the favorable evolution of metabolic disorders and renal function, the patient presented a neurovegetative state due to the initial brain damage.

**Fig. I:**
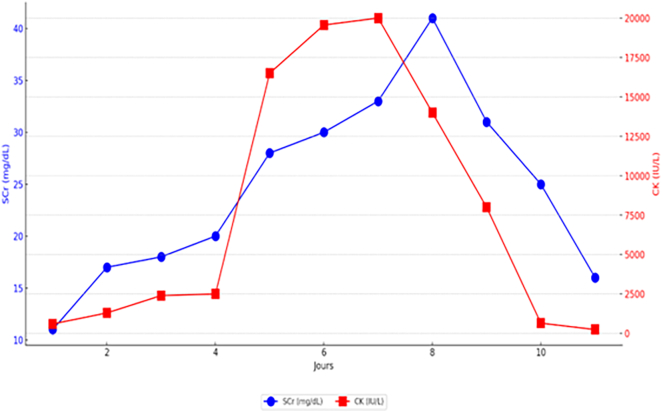
Daily changes in serum creatinine (SCr) and creatine kinase (CK) levels during the treatment of rhabdomyolysis-associated acute kidney injury.

## Discussion

Hypernatremia is a common and serious issue in intensive care, especially among patients with trauma. It occurs in over half of these cases and significantly raises the risk of death [5,7]. The condition results from increased extracellular fluid osmolality, which draws water out of cells, leading to dehydration and shrinkage, particularly in neurological cells.

In trauma brain injured patients, hypernatremia often stems from limited water intake (as seen in comatose individuals), kidney water loss (such as from diabetes insipidus or osmotic diuresis), or losses outside the kidneys (fever or gastrointestinal fluid loss). In this case, the likely cause was kidney water loss due to mannitol use, possibly worsened by fever and excessive intake of serum NaCl 0,9%.

Hypernatremia linked to rhabdomyolysis is rare, especially when combined with kidney failure [8,9]. Imbalances like low potassium and phosphate, along with acidosis and high sodium and osmolality levels, can disrupt sodium movement out of muscle cells. This affects membrane potentials, which are key to preventing muscle breakdown. In exertional rhabdomyolysis, intense activity depletes muscle glycogen, leading to cell damage. Our patient also had an infection, which likely contributed. Infections can raise tumor necrosis factor and interleukin-1, both of which promote muscle breakdown and impair membrane function [11].

When energy production falls short, muscle cells compete for energy-rich phosphate compounds. This limits the energy available to maintain membrane integrity, making cells more permeable and prone to injury. As a result, components like potassium, myoglobin, and creatine kinase (CK) are released into the bloodstream.

The link between hypernatremia and rhabdomyolysis isn't fully understood, but several factors may be involved [[16], [17], [18], [19]]. Severe brain injury, altered mental status, seizures, and hyperosmolar conditions all increase the risk. Diagnosis often includes high CK levels, electrolyte disturbances, and dark urine from myoglobinuria [12]. Causes of rhabdomyolysis vary and include crush injuries, immobilization, toxins, heat, metabolic issues, and electrolyte imbalances. In some cases, fewer than 10%, no clear cause is found [13].

One theory is that hypernatremia inhibits sodiumpotassium pumps in a hyperosmolar environment, disrupting sodiumcalcium exchange [20]. This raises intracellular calcium, which can harm muscle cells by causing constant contraction, activating phospholipase A2, and generating free radicals [21]. On the other hand, rhabdomyolysis itself may worsen hypernatremia [22], creating a vicious cycle.

Our patient had reduced consciousness and was immobile for a prolonged time, both risk factors for rhabdomyolysis. Immobility increases pressure in muscles, reduces blood flow, and leads to injury. Mannitol use likely intensified the hyperosmolar state [10]. The patient received 20% mannitol at 0,5g/kg, which cause dehydration and raise serum sodium and osmolality.

Rhabdomyolysis severity ranges from mild, with elevated muscle enzymes, to severe, with serious electrolyte disturbances, kidney damage, and high mortality. Acute kidney injury (AKI) is a common complication, occurring in 7–10% of cases [14]. Factors like fluid loss, increased abdominal pressure, sepsis, and rhabdomyolysis all contribute. In isolated traumatic brain injury (TBI), AKI affects around 10.6% of patients and is linked to worse outcomes, especially with hemodynamic instability, rhabdomyolysis, or blood transfusions [15]. These risks highlight the importance of early detection and management of fluid and electrolyte imbalances in trauma patients.

Treatment focuses on the underlying cause. For patients with dehydration, hypernatremia, and hyperosmolarity, especially those receiving osmotic diuretics, close monitoring is essential. Optimal management includes proper hydration and alkaline diuresis. If seizures or muscle stiffness occur, anticonvulsants or muscle relaxants may be used. In cases of kidney failure, dialysis may be required, especially with hyperkalemia, uremic symptoms, or fluid overload.

## Conclusion

This case illustrates that severe hypernatremia in patients with traumatic brain injury may induce rhabdomyolysis as a consequence of hyperosmolarity. This hypernatremia is often multifactorial. Early diagnosis and careful management of this electrolyte imbalance are crucial to improve prognosis in these critical cases.

## CRediT authorship contribution statement

**L. Bennis:** Writing – original draft. **Y. Elouardi:** Writing – review & editing, Conceptualization. **I.E. Raihani:** Resources. **I. Oussayeh:** Methodology. **M. Khallouki:** Supervision, Validation.

## Funding sources

The authors received no external funding for this study.

## Declaration of competing interest

The authors declare that they have no known competing financial interests or personal relationships that could have appeared to influence the work reported in this paper.
